# Pathways to Anxiety and Depression in Autistic Adolescents and Adults

**DOI:** 10.1155/2023/5575932

**Published:** 2023-10-03

**Authors:** Amanda L. Richdale, Lauren P. Lawson, Alexa Chalmers, Mirko Uljarević, Eric M. J. Morris, Samuel R. C. Arnold, Julian N. Trollor

**Affiliations:** ^1^Olga Tennison Autism Research Centre, School of Psychology and Public Health, La Trobe University, Melbourne, Australia; ^2^Department of Psychology, Counselling and Therapy, School of Psychology and Public Health, La Trobe University, Melbourne, Australia; ^3^Department of Psychiatry and Behavioral Sciences, School of Medicine, Stanford University, Stanford, CA, USA; ^4^School of Psychology, Western Sydney University, Sydney, Australia; ^5^Translational Health Research Institute, Western Sydney University, Sydney, Australia; ^6^Department of Developmental Disability Neuropsychiatry, Discipline of Psychiatry and Mental Health, UNSW Medicine & Health, University of New South Wales, Sydney, Australia

## Abstract

Individuals diagnosed with autism spectrum disorder (autism) commonly experience co-occurring anxiety and depression, which are significantly associated. These mental health conditions are also variously associated with increased autistic traits, insomnia, intolerance of uncertainty (IU), sensory sensitivity, and autonomic symptoms. However, no research has explored the relationships between IU, sensory sensitivity, autonomic symptoms, insomnia, and autistic traits and how they might be associated with anxiety and depression in autism. This study took a transdiagnostic approach to explore the relationships between anxiety, depression, autistic traits, insomnia, IU, sensory sensitivities, and autonomic arousal in 222 autistic people aged 15-80 years (55.7% female) using path analysis. Four plausible, theoretical models were tested, with model 1 providing best fit and explaining 48% of variance in depression, 37% of variance in anxiety, and 29% of variance in insomnia. Autistic traits and IU were directly associated with anxiety and indirectly associated with depression through anxiety. Anxiety, insomnia, IU, and sensory sensitivity were all directly associated with depression; autonomic arousal and sensory sensitivity were also indirectly associated with depression through insomnia. Thus, multiple pathways can lead to anxiety and depression in autism, not only via known paths such as insomnia and IU but additionally via autonomic arousal and sensory sensitivities which are also elevated in autism. These findings suggest that careful clinical evaluation and individualised treatment plans are needed for autistic adults with anxiety or depression. When considering prevention, programs that help autistic adults reduce arousal, maintain good sleep, and reduce IU may prove fruitful.

## 1. Introduction

Autism spectrum disorder (autism) is characterised by difficulties in social interaction and communication, repetitive behaviours, restricted activities or interests, and atypical sensory features [1] and has a prevalence of about 2% in children and adults [2]. In autistic children, multiple co-occurring conditions are the norm [3], and physical and mental health conditions are more common in autistic than non-autistic adults [4].

Autistic individuals are at increased risk for experiencing anxiety and depression [5], poor sleep quality (hereafter referred to as insomnia) [6], gastrointestinal (GI) symptoms [7], and cardiovascular disease (CVD; [8]). Lifetime rates of anxiety and depression are 42% and 37%, respectively, for autistic adults [5], up to 89% of autistic adults report insomnia symptoms [9, 10], and GI disorders and CVD are reported for 37.4% and 37%, respectively, of autistic adults [4]. These co-occurring conditions can contribute to unemployment [11, 12] and reduced quality of life [10, 13]. Additionally, co-occurring conditions are associated with increased autistic traits in both general and autistic populations [14, 15], and a range of somatic complaints has been shown to positively relate to anxiety, depression, and autistic traits in autistic adults [16].

The significant interrelationships between co-occurring conditions and their association with increased autistic traits indicate that there is merit in investigating models of transdiagnostic factors that may explain these interrelationships. Transdiagnostic factors are mechanisms or conditions that are common across a range of psychiatric disorders and may thus be a focus of treatment [17]. Such models may improve our understanding of the development and maintenance of anxiety and depression in autism, which is critical to targeting effective support for autistic adults. Here, we examine evidence that insomnia, intolerance of uncertainty (IU), autonomic symptoms, and sensory sensitivity may act as transdiagnostic factors in the development of anxiety and depression and that examining these interrelationships may prove fruitful for understanding the development and maintenance of anxiety and depression and targets for mental health support.

Traditionally, insomnia has been considered as a symptom of anxiety and depression rather than as a co-existing disorder [18]. However, recent theories posit insomnia as a causal, transdiagnostic factor for anxiety and depression [19, 20]. Consistent with this view, insomnia, anxiety, and depression have been shown to be reciprocally related [21] and insomnia is a risk factor for later mental ill health [22]. More recently, a network analysis in adults with insomnia (*N* = 1711) showed that congruent with a transdiagnostic perspective, insomnia, anxiety, and depressive symptoms were interrelated [23], while in a second study, insomnia mediated the relationship between anxiety and depressive symptoms in 290 adults with insomnia [24]. In autistic adults (*n* = 220), insomnia symptoms related more strongly to lifetime risk for poor mental health and reduced wellbeing than in a matched group of adults (*n* = 2200) from the general population [25]. Furthermore, improving sleep can result in improved mental health [26]. Autistic adults (*N* = 8) receiving acceptance and commitment therapy for insomnia showed improvements in both insomnia and anxiety [27], while by parent report, autistic adolescents (*N* = 18) receiving behavioural treatment for insomnia also showed improvements in behavioural symptoms including anxiety, depression, and somatisation [28]. Thus, rather than being a symptom of anxiety or depression, insomnia is better conceptualised as a transdiagnostic condition and potential precursor to anxiety and/or depression or mediator of the anxiety-depression relationship.

Intolerance of uncertainty (IU) is a dispositional and negative response to events that are unknown, causing excessive worry [29, 30] and increased negative thoughts [30]. It is transdiagnostic with respect to anxiety, depression [31, 32], and insomnia [31]. In non-autistic, undergraduate students (*N* = 300), IU was significantly associated with anxiety sensitivity (fear of symptoms of anxiety), trait anxiety, depression, and insomnia, with effects on anxiety, depression, and insomnia occurring indirectly through anxiety sensitivity [31], and in non-autistic adolescents (*N* = 2286), worry mediated the relationship between insomnia and IU [33]. Intolerance of uncertainty was correlated with both anxiety and depression in autistic young adults (*N* = 62) [34] and anxiety and sensory sensitivity in both autistic (*n* = 176) and non-autistic adults (*n* = 116) [35]. Cognitive pre-sleep arousal associated with sleep anxiety contributes to insomnia [36], and it may be associated with IU. Autistic adolescents and adults report significantly higher cognitive pre-sleep arousal than non-autistic individuals [37, 38], and improved sleep quality in autistic adults during COVID-19 was associated with concomitant reductions in cognitive pre-sleep arousal [39]. However, the relationship between IU and insomnia in autism has not yet been explored. Thus, IU may be a critical transdiagnostic factor for the development and maintenance of anxiety, depression, and insomnia.

Sensations associated with somatic arousal are related to autonomic responses associated with experiences of internal and external stimuli [40] and may indicate autonomic dysfunction. There is emerging evidence that autonomic dysfunction is associated with psychiatric diagnoses [41] and insomnia [8]; individuals with high somatic arousal are likely to have clinical anxiety [42, 43] or depression [42], and somatic pre-sleep arousal is a causal factor for insomnia [43, 44]. Autonomic dysfunction can be associated with a range of physical health conditions including those related to the cardiovascular system, temperature regulation, orthostatic responses, visual responses [45, 46], and GI conditions [45–47]. Young adult psychiatric patients (*N* = 491) reported elevated GI symptoms compared with controls, with 89.4% also having a lifetime depressive episode and 61.2% having an anxiety disorder [46]. Further, treating panic disorder in those with co-occurring GI symptoms led to reductions in these symptoms [48]. Increased CVD risk, abnormal pupillary responses, orthostatic intolerance, and differences in thermoregulation are also reported in autism [4, 7, 41, 49, 50], and GI symptoms have been related to poor mental health in autistic adults and non-autistic adults with high autism traits [15]. Overall, these findings suggest that autonomic dysfunction may also be transdiagnostic for mental health conditions and insomnia.

Sensory sensitivity refers to hyper- or hypo-reactivity to sensory stimuli across sensory modalities and is common across all sensory modalities in those with high autistic traits, but more variable across sensory modalities in low-autistic trait individuals [51]. In non-autistic populations, sensory sensitivity is shown to relate with anxiety, depression, and insomnia [52–54]. In autistic children, sensory over-responsivity was associated with anxiety and insomnia and suggested to be due to hyperarousal [55]. Anxiety in autistic adults (*n* = 176) was associated with sensory hyper- and hypo-sensitivity [35], depression was related to sensory hypo-sensitivity in autistic boys [56], and visual sensory hyper-reactivity was related to insomnia in a sample of 631 autistic adults [57]. Further, in both autistic and non-autistic children, there is a relationship between IU and sensory sensitivity which is mediated by anxiety, with the IU and anxiety relationship being stronger in autistic children and accounting for around half the variance in sensory sensitivity in this group [58]. Thus, sensory sensitivity too may be transdiagnostic for anxiety, depression, and insomnia.

In summary, IU is transdiagnostic for anxiety, depression, and insomnia, and insomnia is transdiagnostic for anxiety and depression. Additionally, the research summarised above supports sensory sensitivity, and autonomic symptoms as potential transdiagnostic factors for anxiety, depression, and insomnia in both autistic and non-autistic populations. Furthermore, autistic traits are continuous in the general population [59], with increased autistic traits conferring increased likelihood for anxiety, depression, and insomnia and increased autonomic symptoms and sensory sensitivity. Understanding how these transdiagnostic factors and autistic traits may influence anxiety and depression in autistic adults, within a single model, can offer unique insights into the prevention and treatment of anxiety and depression by identifying potential points for support.

## 2. The Current Study

**Figure 1:**
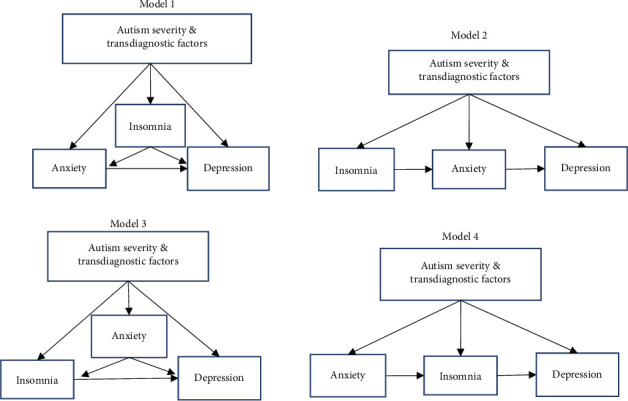
Models for investigation of interactions between transdiagnostic factors (IU, arousal, and sensory sensitivity) and insomnia, anxiety, and depression. Model 1: insomnia is associated directly with anxiety and depression, while anxiety also partially mediates the relationship between insomnia and depression. Model 2: anxiety mediates the relationships between insomnia and depression. Model 3: anxiety is associated directly with insomnia and depression, while insomnia also partially mediates the relationship between anxiety and depression. Model 4: insomnia mediates the relationship between anxiety and depression.

Our aim was to provide an initial characterisation of the inter-relationship between anxiety and depression, transdiagnostic factors (insomnia, IU, sensory sensitivity, and autonomic symptoms), and autistic traits among older autistic adolescents and autistic adults. To our knowledge, no study has examined how these transdiagnostic factors, together with autistic traits, may act as predictors for anxiety and depression in autism within a single model. Four theoretically plausible models describing these relationships were proposed (Figure 1) and were tested using path analysis. Three research questions were posed:

1. Do autism trait severity, autonomic arousal, sensory sensitivity, IU, and insomnia contribute directly and/or indirectly to anxiety and depression? It was hypothesised that these variables would have a significant positive relationship with anxiety and depression and that anxiety would be positively related to depression
2. Does insomnia act as a mediator in relationships between IU, sensory sensitivity, autonomic arousal, and autistic traits and depression and anxiety?
3. Does anxiety act as a mediator in the relationships between autonomic arousal, sensory sensitivity, IU, and autistic traits and insomnia and depression?

## 3. Method

### 3.1. Participants

Participants were from the baseline phase of the longitudinal Study of Australian School Leavers with Autism (SASLA) [60] and the Australian Longitudinal Study of Autism in Adulthood (ALSAA) [61]. The current sample consisted of 222 autistic older adolescents and adults, age range 15-80 years. All participants had a self-reported diagnosis of autism, and 55.7% (*n* = 117) of the sample was female.

### 3.2. Materials

#### 3.2.1. Demographics

Participants reported their age, gender, details of their autism diagnosis, education, and employment, whether they had been diagnosed with an internalising disorder (anxiety, depression) and/or an externalising disorder (attention-deficit/hyperactivity disorder (ADHD)), and current medication use.

The *Autism Spectrum Quotient-Short* (AQ-Short) *form* [62]) is a 28-item self-report measure of autistic traits. Items are scored on a 4-point Likert scale ranging from *definitely agree* to *definitely disagree* where higher scores indicate more severe symptoms of autism. It contains no questions pertaining to sensory sensitivities. The AQ-Short has acceptable to good internal consistency with internal coefficient alpha ranging from .77 to .86 (*α* = .85 in the current sample).

The *Pittsburgh Sleep Quality Index* (PSQI) [63] was used as a measure of insomnia. It is an 18-item self-report questionnaire assessing sleep over the past month across seven areas: sleep duration, sleep disturbance, sleep latency, daytime dysfunction, sleep efficiency, sleep quality, and sleep medication. A total sleep quality score is calculated from the seven subscale scores (range 0 to 21) where a score > 5 indicates poor sleep. The PSQI correlates highly (*r* = .80) with the insomnia severity index [64] and has high coefficient alpha (.83 for the overall scale). It has been used previously in autistic adults [10, 65] with *α* = .73 in the current sample.

The *DSM-5-Dimensional Anxiety Scale for Generalised Anxiety Disorder* (DSM-GAD) [66] consists of 10 self-report items measured using a 5-point Likert scale. An average item score which ranges from 0 (*no anxiety*) to 4 (*extreme anxiety*) is calculated from the total score. The measure for DSM-GAD has good test-retest reliability and internal consistency [67] with coefficient alpha ranging from .85 to .92; in the current sample, *α* = .90.

The *Patient Health Questionnaire* (PHQ-9) [68] is a self-report measure of the severity of depressive symptoms over the past two weeks. It has 9 items measured on 4-point Likert scale ranging from 0 (*not at all*) to 3 (*nearly every day*), with a total score ≥ 10 being indicative of depression. The PHQ-9 has good internal consistency and test-retest reliability and has been validated for use with autistic adults with *α* = .91 [69]; in the current sample, *α* = .90.

The *Intolerance of Uncertainty Scale, Short-Form* (IUS-12) [29] is a 12-item questionnaire which measures the degree to which individuals find unpredictable and negative events unacceptable with higher scores indicating increased IU. Items are measured on a 5-point scale varying from 1 (*not at all characteristic of me*) to 5 (*entirely characteristic of me*). The IUS-12 has good test-retest reliability and coefficient alpha (.91); in the current sample, *α* = .93.

The *Glasgow Sensory Questionnaire* (GSQ) [70] measures sensory sensitivity to stimuli. It is a 42-item self-report measure with questions across seven areas of hyper- and hypo-sensory sensitivities: visual, auditory, gustatory, olfactory, tactile, vestibular, and proprioception. Sensitivity to each item is asked using a 5-point scale ranging from *never* to *always*, with higher scores indicating increased sensory sensitivities. It is strongly associated with increased autistic traits in adults, with no reported male/female difference, and *α* = .94 [70]. It has been validated in autistic adults with *α* = .91 [71]; in the current sample, *α* = .93.

The *Composite Autonomic Symptom Score-31* (COMPASS-31) [45] is a 31-item self-report questionnaire assessing autonomic nervous system function by measuring the severity of autonomic symptoms across six domains: orthostatic intolerance, vasomotor, secretomotor, gastrointestinal, constipation, bladder, and pupillomotor. Participants are asked if they experience symptoms and then questions related to symptom frequency and severity. Total scores range from 0 to 100, with high scores representing more severe symptoms. A global autonomic symptom severity score is calculated from sum of the weighted scores for each domain. Poor to excellent internal validity was reported for each of the six weighted domain scores with coefficient alpha ranging from .48 for to .92 [45]. Coefficient alpha for the total score in this sample, using the six weighted domain scores, was *α* = .51.

### 3.3. Procedure

Ethics approvals for the SASLA and ALSAA studies were obtained from the La Trobe University (HEC14-095) and University of New South Wales Human Research Ethics Committees (HC15001), respectively. For both studies, participants were recruited Australia-wide via autism support groups and associations, online recruiting websites, advertising, and word of mouth. Participants who expressed interest were sent information about the study and provided informed consent prior to participation. Parents also provided consent for those <18 years. After receiving their consent, a unique link to the online survey was sent to participants; a hard copy survey was available on request. Both SASLA and ALSAA participants completed the survey on entry to the study (baseline) and 2 years later; SASLA participants also completed a survey at 1 year. All measures in this study were only available in the baseline surveys. The COMPASS and IU scales were not included in the ALSAA 2-year survey, and the GSQ was not included in either longitudinal survey at 2 years.

### 3.4. Analysis

Descriptive statistics were used to summarise sample characteristics. Assumptions of linear regression (outliers, linearity, normality, multicollinearity, and homoscedasticity) were tested. A path analysis was run for each of the four proposed models using Mplus [72] to describe the relationship that transdiagnostic factors (insomnia, IU, sensory sensitivities, and autonomic function) and autism trait severity have with anxiety and depression in autistic adolescents and adult (see Figure 1). Based on the recommendations from Wolf et al. [73], the sample should include a minimum of 10 participants per variable; the current models contain seven variables, thus requiring at least 70 participants. Maximum likelihood was used to test the models. Bootstrapping with 1000 samples was used due to the moderate sample size, and it is suggested to be used when testing direct and indirect relationships between variables [74]. All correlations between age and the other variables were either not significant or weak (Table 1); thus, age was not included as a covariate in the model. However, there were significant differences between male and female participants, such that females had higher scores on autism traits, IU, autonomic arousal, sensory sensitivity, insomnia, anxiety, and depression measures (Table 2). As such, sex was included as a covariate in the models. Model fit was measured via the chi-square with non-significant values indicating good fit. Standardised root mean square residual (SRMR; value less than .08 indicating model fit), root mean square error of approximation (RMSEA; value less than .05 indicating model fit), comparative fit index (CFI; value higher than .95 indicating model fit), and the Tucker-Lewis index (TLI; value higher than .90 indicating model fit) were also used to assess model fit.

## 4. Results

Participant characteristics and mean scores for autistic traits, IU, autonomic symptoms, sensory sensitivity, insomnia, anxiety, depression, and age can be found in Table 2. The four most frequent self-reported psychiatric diagnoses were anxiety, depression, ADHD, and OCD, with over half of the participants reporting a current anxiety diagnosis and about 40% reporting depression; about a third of the sample was currently on antidepressant medication (Table 1). While there were medium to strong positive correlations found between autism trait severity, sensory sensitivity, autonomic arousal, IU, insomnia, anxiety, and depression, all correlations were <.6, with the strongest correlation being between anxiety and depression.

### 4.1. Path Analysis

For each model, analyses were first conducted using a fully saturated model (df = 0). Nonsignificant pathways and paths with coefficients < .10 were removed. Bootstrapping was performed with 1000 samples with 95% confidence intervals computed. Based on the model fit statistics, model 1 was the best fit to the data, meeting acceptable criteria on all four model fit indices. See Table 3 for model fit statistics for the four trimmed models.

**Figure 2:**
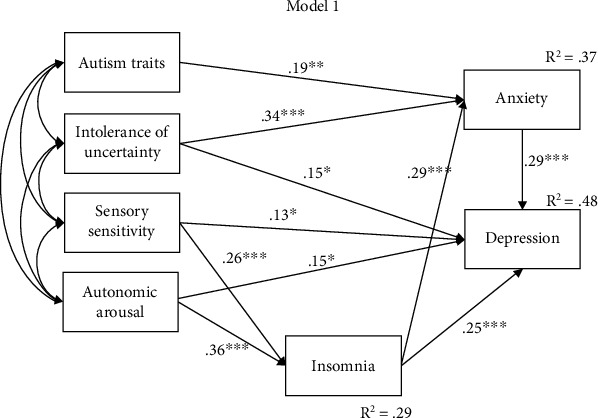
Trimmed model 1. Path analysis between transdiagnostic factors and insomnia, anxiety, and depression, controlling for sex. Standardised estimates and squared multiple correlation (*R*^2^) are shown. ⁣^∗^*p* < .05; ⁣^∗∗^*p* < .01; ⁣^∗∗∗^*p* < .001.

Model 1 explained 29% of the variance in insomnia, 37% of the variance in anxiety, and 48% of the variance in depression symptoms. Autism traits were significantly positively associated with anxiety (95%CI = .068, .226). IU was significantly positively associated with anxiety (95%CI = .199, .373) and depression (95%CI = .029, .170). Sensory sensitivity was significantly positive associated with insomnia (95%CI = .025, .061) and depression (95%CI = .009, .064). Somatic arousal was directly related to insomnia (95%CI = .067, .127) and depression (95%CI = .021, .117). Insomnia was positively related to anxiety (95%CI = .387, .807) and depression (95%CI = .252, .584), and anxiety was directly related to depression (95%CI = .128, .323). The trimmed model for model 1 is shown in Figure 2. The trimmed models for models 2, 3, and 4 can be found in Supplementary Figures S1-S3.

### 4.2. Indirect Effects of Model 1

Indirect and total effects for model 1 are presented in Table 4. Autism traits, IU, and insomnia all had significant indirect effects on depression via anxiety. Sensory sensitivity and somatic arousal had significant indirect effects on depression through two pathways: (1) via insomnia and (2) via insomnia then anxiety.

## 5. Discussion

Our aim was to explore the relationships between four transdiagnostic factors (insomnia, IU, sensory sensitivity, and autonomic symptoms), autistic traits, and anxiety and depression among autistic adolescents and adults via correlation and path analysis. In support of our hypotheses, all transdiagnostic factors and autistic traits had a significant, positive relationship with each other and with anxiety and depression. Of the four theoretical models tested, the best fit model, model 1, accounted for over a third of the variance in anxiety symptoms, half the variance in depressive symptoms, and close to a third of variance in insomnia symptoms. Autistic traits, IU, and insomnia contributed directly to the severity of anxiety symptoms; all four transdiagnostic factors and anxiety contributed directly to the severity of depressive symptoms; autistic traits and IU contributed indirectly to depression through anxiety; and sensory sensitivities and autonomic symptoms contributed to depression indirectly through insomnia. Anxiety fully mediated the relationship between autistic traits and depression and partially mediated the relationship between (1) IU and depression; (2) sensory sensitivity, insomnia, and depression; and (3) somatic arousal, insomnia, and depression. Further, insomnia partially mediated the relationships between sensory sensitivity and somatic arousal on depression. These findings support the wide range of previous studies showing various correlations and predictive relationships between anxiety, depression, autistic traits, insomnia, IU, sensory sensitivity, and autonomic symptoms in autism as summarised in our introduction. A diagnosis of autism increases the likelihood that an individual will experience clinical anxiety and/or depression markedly, potentially by virtue of the noted elevation of these transdiagnostic risks factors in autism compared with individuals in the general population.

Sensory sensitivity and autonomic symptoms directly influenced depression as well as having indirect pathways through insomnia to both anxiety and depression. Sensory sensitivity and autonomic symptoms are indicators of physiological or somatic arousal. It has been previously suggested that hyperarousal associated with sensory sensitivity and anxiety is implicated in poor sleep in autistic children [55, 75, 76], while Bitsika et al. [56] reported that sensory avoidance was strongly associated with depression in autistic boys. GI symptoms in particular have been related to insomnia [15]. In non-autistic adults (*N* = 176), sensory hypersensitivity was higher in poor sleepers than good sleepers, and regression analyses showed that sensory sensitivity, depressive symptoms, and anxious temperament significantly predicted poor sleep [52]. Our findings are consistent with the physiological hyperarousal theory of insomnia [44, 77] and the finding that somatic pre-sleep arousal was associated with insomnia symptoms in autistic adults [37]. The relationship between sensory sensitivity, autonomic symptoms, and insomnia and their impact on mental health are also compatible with autonomic hyperarousal theories of autism [78, 79].

While we did not have a direct measure of cognitive arousal, IU is related to negative beliefs about unpredictable events and associated with psychological inflexibility, anxiety sensitivity, and pathological worry, all of which increase both cognitive and physiological arousal [32]. Consistent with the previous research in both non-autistic and autistic samples, IU had a direct effect on both anxiety and depression, but surprisingly not insomnia. While we found a significant, moderate correlation between IU and insomnia, based on the cognitive arousal theory of insomnia, we had expected to find either a direct or indirect relationship between these variables within our model. Previous work in non-autistic adults has found that IU mediates the relationship between worry and insomnia [33] and cognitive hyperarousal was associated with insomnia, while somatic hyperarousal was predictive of anxiety [43]. In autistic adolescents, both pre-sleep cognitive and somatic arousal were associated with poor sleep [38], but in adults, while both were high, somatic but not cognitive pre-sleep arousal was associated with sleep [37]. Thus, IU appears more salient with respect to anxiety than insomnia in autism, or it may be that IU is simply a poor indicator of cognitive arousal associated with sleep. Overall, our results suggest that patterns of association between hyperarousal variables and insomnia differ in autism compared to non-autistic populations, with physiological arousal being more salient for insomnia than cognitive arousal.

Insomnia has the strongest genetic and phenotypic overlap with anxiety and depression [80], which is consistent with our model showing inter-relationships between anxiety, depression, and insomnia in autistic adults. Additionally, there is a lack of significant genetic [80, 81] or phenotypic [80] overlap between insomnia and autism. This, together with our findings, suggests that risk for insomnia symptoms in autism may be driven by intrinsic arousal factors that increase risk for mental health concerns and poor sleep. This may be compatible with a recent theoretical perspective that rather than being due to difficulties with circadian or homeostatic sleep control, insomnia has its roots in emotionally stressful experiences or trauma, which may have occurred in early childhood or more recently and which promote nighttime cognitive and physiological hyperarousal [80]. Consistent with this view, self-injurious behaviour, which can be a signal of distress, and sensory sensitivity predicted future sleep difficulties in autistic children [82]. Elevated stress and exposure to traumatic events, particularly in relation to social encounters, are reported by autistic adults [83] and can increase vulnerability to mental health concerns and insomnia. These avenues may be fruitful for the future exploration of the increased susceptibility to insomnia symptoms and mental health concerns in autism.

### 5.1. Clinical Implications

Clinically, our model suggests multiple points for intervention for depression in autistic adults, including addressing IU, physiological arousal, insomnia, and anxiety. Insomnia, in particular, difficulty with sleep initiation, predicted the onset of major depression in a 6-year follow-up of non-autistic adults from the Netherlands Study of Depression and Anxiety (*N* = 768) [84], while treating insomnia using CBT-I in those with both depression and insomnia is associated with improvements in depression and potential for improved depression outcomes [85–87]. Thus, it may be essential to treat insomnia as well as depression in those with both conditions. Treating insomnia in those with co-occurring anxiety may also improve treatment outcomes [87], while treating anxiety can lead to modest improvement in insomnia [88], including in autism [89, 90]. Additionally, depression and insomnia are risk factors for suicidal ideation and suicide [20, 91]. Depression is also a risk factor for suicidal ideation in autistic individuals [92] and suicide risk is increased in autism compared with the general population [93], further illustrating the importance of taking a transdiagnostic view to intervention for autistic adults.

The findings that somatic arousal, sensory sensitivity, and IU are underlying mechanisms for anxiety, depression, and insomnia in autism, either directly or indirectly, have important clinical implications. The significance of these relationships suggests that developing treatments that reduce somatic arousal, sensory sensitivity, and/or IU may be beneficial in treating these disorders. More specifically, for individuals experiencing symptoms of anxiety and/or insomnia, addressing somatic arousal may be the first point for intervention and may improve these symptoms, in turn reducing symptoms of depression or minimising the risk of later developing depression. Additionally, these results suggest that using techniques specific to decreasing somatic symptoms such as deep breathing and progressive muscle relaxation could be beneficial to implement early in treatment [43]. The current research suggests that treating insomnia or psychopathology as separate conditions, or without addressing common transdiagnostic factors, would lead to autistic adolescents and adults being less likely to experience positive treatment outcomes.

### 5.2. Strengths and Limitations

A major strength of this study was the exploration of four plausible, theoretical models allowing us to test for the first time the relationships between autistic traits, sensory sensitivity, autonomic arousal, IU, insomnia, anxiety, and depression, all of which have consistently and variously been shown to be interrelated in the current literature, using standardised questionnaires. Additionally, the PHQ-9 and GSQ have been previously validated for use in autistic adults [69, 71], and the PSQI has been used previously in autistic adult samples [10, 65]. Third, we had a large sample of autistic individuals with no intellectual disability representing a wide age range from late adolescence to old age. Finally, the fit for model 1 was good based on chi-square and all other model fit indices. This gives confidence that this model deserves further exploration and may be used to guide future prevention and intervention strategies for anxiety and depression. However, some limitations need to be considered.

First, there is construct overlap with significant associations between all measures in the models tested (range: *r* = .23 to *r* = .57), which, based on existing literature and diagnostic criteria for anxiety and depression, is expected. Nevertheless, the DSM-GAD contains two questions which refer to autonomic symptoms (e.g., *sweaty*, *felt racing heart*, and *felt tense muscles*): one of which also includes *trouble sleeping* and the PHQ-9 includes the following question: *Trouble falling asleep, staying asleep or sleeping too much*. While there is some overlap with other questionnaires, multiple symptoms are referred to in each question and these symptoms form part of DSM diagnostic criteria for these disorders. Therefore, they were retained in the analyses but may have inflated some associations.

Second, the PSQI, while correlating highly with measures of insomnia, is a measure of overall sleep quality as it also includes questions associated with other aspects of poor sleep. In a model examining relationships between IU, anxiety sensitivity, trait anxiety, depression, and sleep, sleep was measured using both the insomnia severity index [94] and the PSQI, and the ISI was found to relate more closely to anxiety, while the PSQI was more closely associated with depression [31]. Thus, the choice of sleep measure may have had some effect on our model outcomes.

Third, our sample may not be representative of all autistic individuals. Those with an ID were not included in our analyses, and just over half of our sample was female, exceeding the reported adult male : female ratio of 2.57 [95]. A high proportion of females is common in survey research in both autistic (e.g., [96]) and non-autistic samples (e.g., [97]), and in Australia, being female with no ID is associated with later age of diagnosis [98], potentially increasing the number of females in adult compared to child samples. The average difference in age of diagnosis for male and female participants in an Australian sample of autistic people aged from 15 to 80 years (*N* = 657), which included the SASLA and ALSAA participants in this study, was 10 years (females: 33.6 years, males: 24.6 years), with current age being strongly associated with age at diagnosis [98].

Fourth, autism diagnosis was via self-report, though we collected additional diagnostic information (year of diagnosis, presence of diagnostic report, and clinician), as well as copies of diagnostic reports and parent confirmation (SASLA) of diagnosis for participants where possible. Additionally, all ALSAA participants who were identified as autistic but did not report a formal diagnosis were excluded (this group was not included in SASLA).

Finally, while path analysis is designed to test causal pathways, we had a cross-sectional data set. We tested four theoretically plausible models, only one of which was supported, which gives some confidence in our findings. However, there are other possible models that could be tested, and we cannot know if our model would hold longitudinally. Furthermore, there are multiple valid measures of the constructs we tested, and the choice of measure may potentially alter model relationships. Future research should confirm the results of this study by examining relationships over time using longitudinal data and other valid measures of the constructs investigated here, which have preferably been validated for autistic individuals.

## 6. Conclusions

Uniquely, we have shown for the first time how autistic traits and transdiagnostic factors associated with autism may both be related to and underpin the high rates of co-occurring anxiety and depression. Understanding why autistic people are at such high risk for anxiety in early childhood and addressing this may be paramount. Developing transdiagnostic approaches to intervention for insomnia, anxiety, and depression is essential.

## Figures and Tables

**Table 1:** Correlations between the autism trait severity, transdiagnostic factors, and insomnia, anxiety, and depression.

	1	2	3	4	5	6	7	8
(1) Autism traits (AQ-Short)	—							
(2) Intolerance of uncertainty (IUS-12)	.47⁣^∗∗^	—						
(3) Autonomic arousal (COMPASS-31)	.23⁣^∗∗^	.26⁣^∗∗^	—					
(4) Sensory sensitivity (GSQ)	.49⁣^∗∗^	.48⁣^∗∗^	.46⁣^∗∗^	—				
(5) Insomnia (PSQI)	.24⁣^∗∗^	.31⁣^∗∗^	.49⁣^∗∗^	.44⁣^∗∗^	—			
(6) Anxiety (GAD)	.42⁣^∗∗^	.52⁣^∗∗^	.30⁣^∗∗^	.42⁣^∗∗^	.44⁣^∗∗^	—		
(7) Depression (PHQ-9)	.32⁣^∗∗^	.47⁣^∗∗^	.45⁣^∗∗^	.50⁣^∗∗^	.55⁣^∗∗^	.57⁣^∗∗^	—	
(8) Age (years)	−.29⁣^∗∗^	−.14	−.04	−.03	−.05	−.34⁣^∗∗^	−.02	—

⁣^∗∗^*p* < .01.

**Table 2:** Participant characteristics, mean (*M*) and standard deviation (SD) for main variables, and comparisons between males and females.

	Total		Male		Female		Comparison
Continuous variables	*N*	*M* (SD)	*n*	*M* (SD)	*n*	*M* (SD)	
Autism traits (AQ-Short)	222	84.75 (11.53)	104	81.86 (12.19)	117	87.36 (10.35)	*t* (219) = 3.63, *p* < .001, *d* = .49
Intolerance of uncertainty (IUS-12)	222	39.35 (10.83)	104	37.50 (11.08)	117	41.11 (10.33)	*t* (219) = 2.51, *p* = .013, *d* = .34
Autonomic arousal (COMPASS-31)	222	23.34 (15.32)	104	19.49 (14.85)	117	26.96 (14.85)	*t* (219) = 3.74, *p* < .001, *d* = .50
Sensory sensitivity (GSQ)	222	70.75 (25.21)	104	62.38 (24.40)	117	78.59 (23.20)	*t* (219) = 5.06, *p* < .001, *d* = .68
Insomnia (PSQI)	222	7.88 (4.11)	104	6.75 (3.37)	117	8.94 (4.40)	*t* (219) = 4.12, *p* < .001, *d* = .55
Anxiety (GAD)	222	10.20 (8.79)	104	8.14 (8.14)	117	12.11 (8.94)	*t* (219) = 3.44, *p* < .001, *d* = .46
Depression (PHQ-9)	222	9.80 (6.92)	104	8.72 (7.00)	117	10.84 (6.68)	*t* (219) = 2.30, *p* = .022, *d* = .31
Age (years)	222	36.10 (15.04)	104	35.86 (17.03)	117	36.45 (13.09)	*t* (219) = .29, *p* = .769, *d* = .04

Categorical variables	*N*	*n* (%)	*N*	*n* (%)	*N*	*n* (%)	
Current mental health condition							
Anxiety	213	125 (58.7)	114	75 (65.8)	98	50 (51.0)	*χ* ^2^ (1) = 4.75, *p* = .029, *Φ* = .15
Depression	214	93 (43.5)	112	54 (48.2)	101	39 (38.6)	*χ* ^2^ (1) = 1.99, *p* = .158, *Φ* = .10
ADHD	201	31 (15.4)	106	15 (14.2)	94	16 (17.0)	*χ* ^2^ (1) = .313, *p* = .576, *Φ* = .04
Obsessive compulsive disorder	203	15 (7.4)	108	11 (10.2)	94	4 (4.3)	*χ* ^2^ (1) = 2.57, *p* = .276, *Φ* = .11
Current medication use							
Sleeping medication	214	18 (8.4)	115	12 (10.4)	99	6 (6.1)	*χ* ^2^ (1) = 1.32, *p* = .250, *Φ* = .08
Anxiety or nerves	214	33 (15.4)	116	17 (14.7)	98	16 (16.3)	*χ* ^2^ (1) = .114, *p* = .736, *Φ* = .02
Tranquilisers	212	2 (0.9)	114	0 (0.0)	98	2 (2.0)	Fishers exact: *p* = .213 (2 sided)
Antidepressants	215	77 (35.8)	116	50 (43.1)	99	27 (27.3)	*χ* ^2^ (1) = 5.82, *p* = .016, *Φ* = .16
Mood stabilisers	214	10 (4.7)	115	5 (4.3)	99	5 (5.1)	Fishers exact: *p* = 1.00 (2 sided)
Other medications	212	81 (38.2)	114	46 (40.4)	98	35 (35.7)	*χ* ^2^ (1) = .480, *p* = .488, *Φ* = .05

**Table 3:** Model fit statistics.

Model	*χ* ^2^ (df)	*p* value	RMSEA	CFI	TLI	SRMR
Model 1	4.360 (5)	**.499**	**<.001**	**1.00**	**1.08**	**.018**
Model 2	21.37 (6)	.002	.108	**.956**	.840	**.033**
Model 3	10.06 (5)	**.073**	**.068**	**.986**	.937	**.034**
Model 4	31.85 (6)	<.001	.140	.926	.730	**.047**

Note: RMSEA = root mean square error of approximation; TLI = Tucker-Lewis index; CFI = comparative fit index; SRMR = standardised root mean square residual. Acceptable values for *p* and fit indices are in bold.

**Table 4:** Indirect and total effects for model 1.

Pathway	*β*	SE	95% CI	*p*
Autism traits ➔ anxiety ➔ depression	.056	.023	[.015, .061]	.016
IU ➔ anxiety ➔ depression	.099	.037	[.038, .160]	.007
Sensory sensitivity ➔ insomnia ➔ depression	.066	.023	[.028, .104]	.005
Sensory sensitivity ➔ insomnia ➔ anxiety ➔ depression	.022	.010	[.005, .038]	.028
Autonomic symptoms ➔ insomnia ➔ depression	.092	.028	[.045, .139]	.001
Autonomic symptoms ➔ insomnia ➔ anxiety ➔ depression	.030	.012	[.011, .050]	.009
Insomnia ➔ anxiety ➔ depression	.083	.028	[.037, .129]	.003
Autism traits total	.056	.023	[.018, .093]	.016
Sensory sensitivity total	.213	.061	[.113, .313]	<.001
Autonomic symptoms total	.272	.062	[.170, .373]	<.001
IU total	.249	.067	[.139, .358]	<.001
Insomnia total	.335	.063	[.232, .438]	<.001

Note: *β* = standardised path coefficient; SE = standard error.

## Data Availability

These data are available from the Autism CRC under license and so cannot be made freely available. Requests for access to these data should be made to the Autism CRC.
