# Supplementary material for: Pathways to Anxiety and Depression in Autistic Adolescents and Adults
Source: Depress Anxiety. 2023 Oct 3;2023:5575932. doi: 10.1155/2023/5575932 (PMC11921850; doi:10.1155/2023/5575932)
Supplement: Supplementary Materials — Figures for trimmed models 2, 3, and 4 can be found in Supplementary Figures S1-S3. [file 5575932.f1.docx]

**Supplementary Figures**

*Figure S1. Trimmed Model 2.* Path analysis between transdiagnostic factors and insomnia, anxiety and depression, controlling for sex. Standardised estimates and squared multiple correlation (R²) are shown. *p < .05; ** p < .01; *** p < .001

*Figure S2. Trimmed Model 3.* Path analysis between transdiagnostic factors and insomnia, anxiety and depression, controlling for sex. Standardised estimates and squared multiple correlation (R²) are shown. *p < .05; ** p < .01; *** p < .001

*Figure S3. Trimmed Model 4.* Path analysis between transdiagnostic factors and insomnia, anxiety and depression, controlling for sex. Standardised estimates and squared multiple correlation (R²) are shown. *p < .05; ** p < .01; *** p < .001
